# Supplementary material for: TRIM28 Regulates Dlk1 Expression in Adipogenesis
Source: Int J Mol Sci. 2020 Sep 30;21(19):7245. doi: 10.3390/ijms21197245 (PMC7582669; doi:10.3390/ijms21197245)

**Table S1.1 Pathway analysis with Trim28 KD vs. control 3T3-L1 at day 0 by using KEGG pathway database**


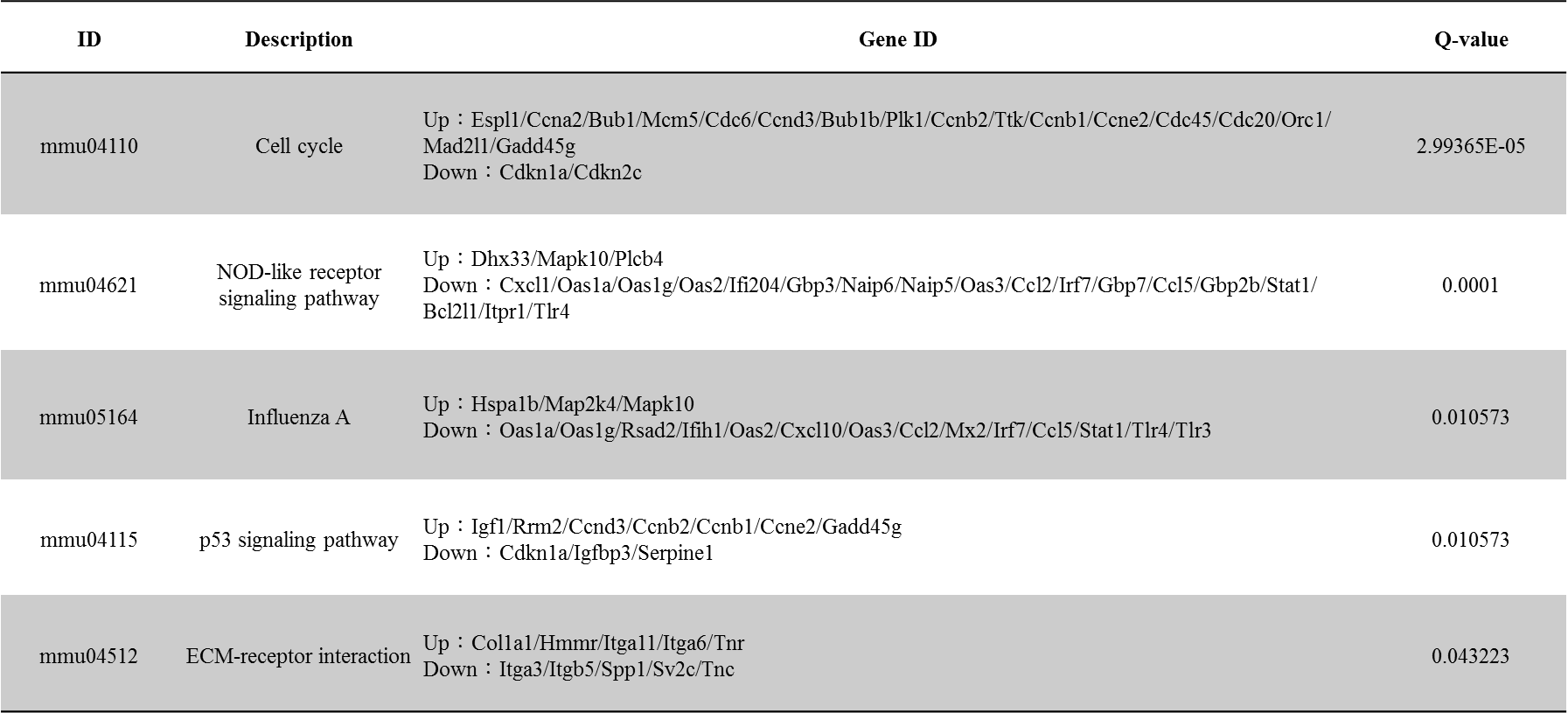


**Table S1.2 Pathway analysis with Trim28 KD vs. control 3T3-L1 at day 2 by using KEGG pathway database**


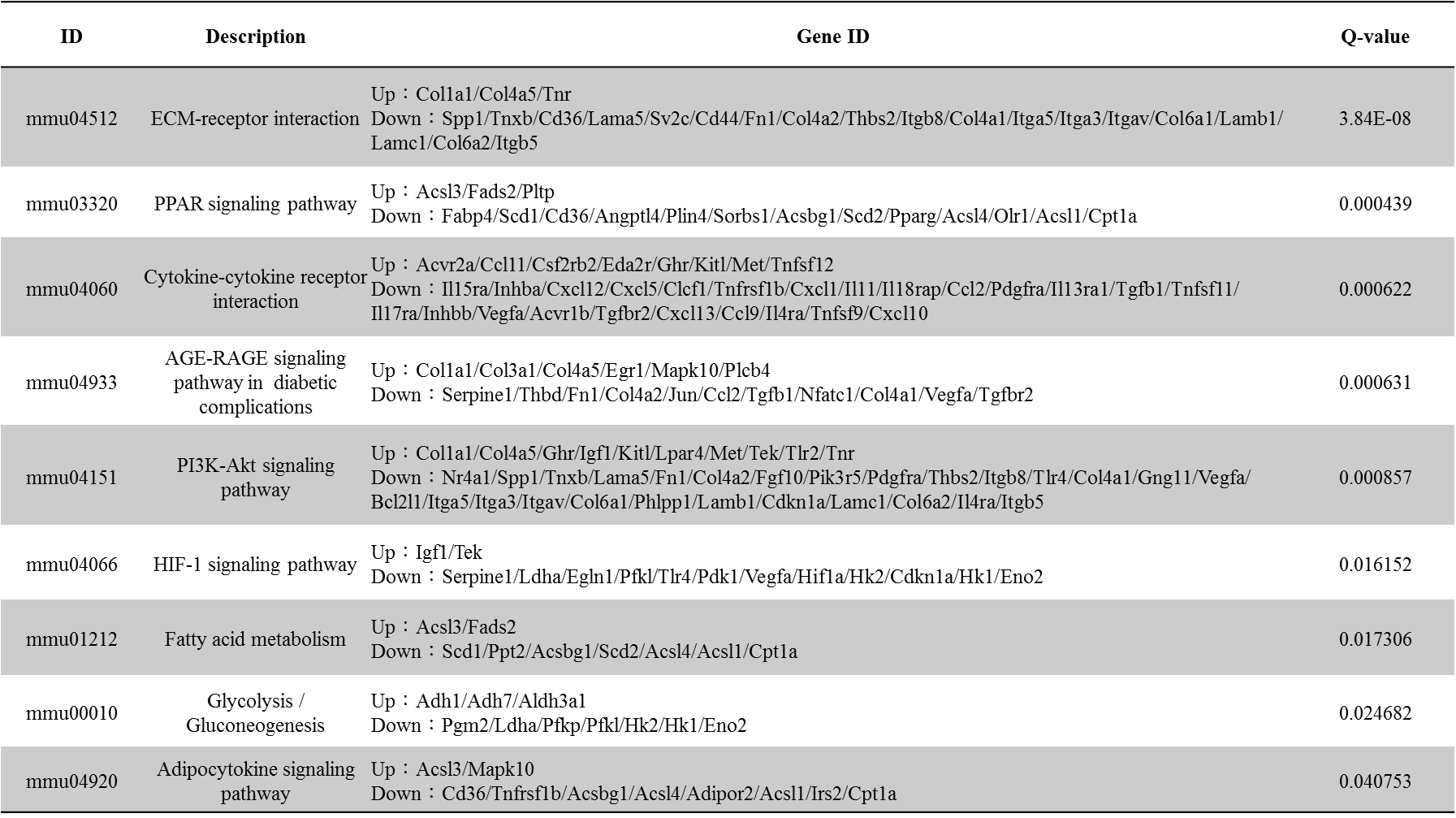


**Table S1.3 Bioinformatics analysis with Trim28 KD vs. control 3T3-L1 at day 0 by using GO database**


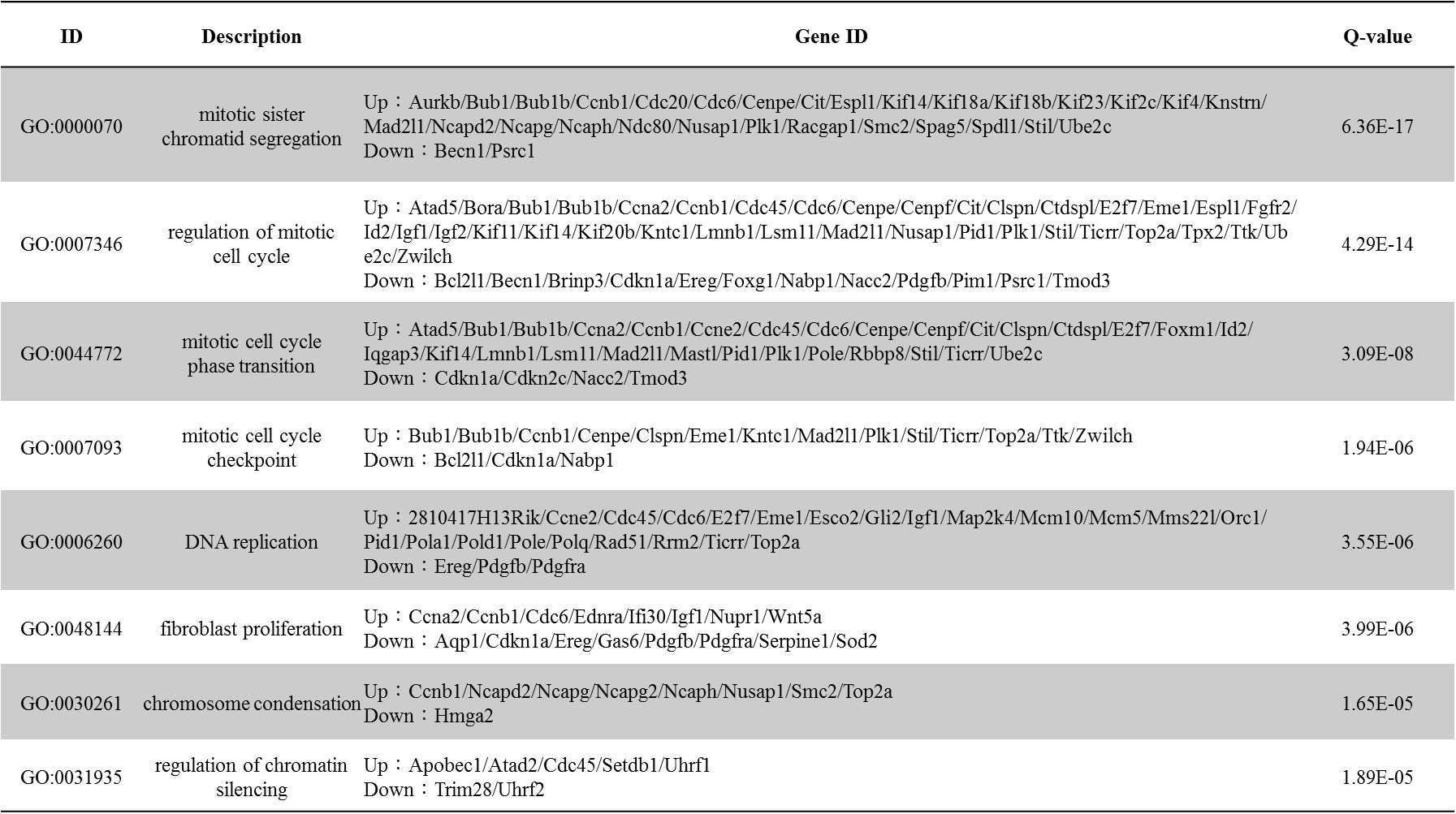


**Table S1.3 continued**


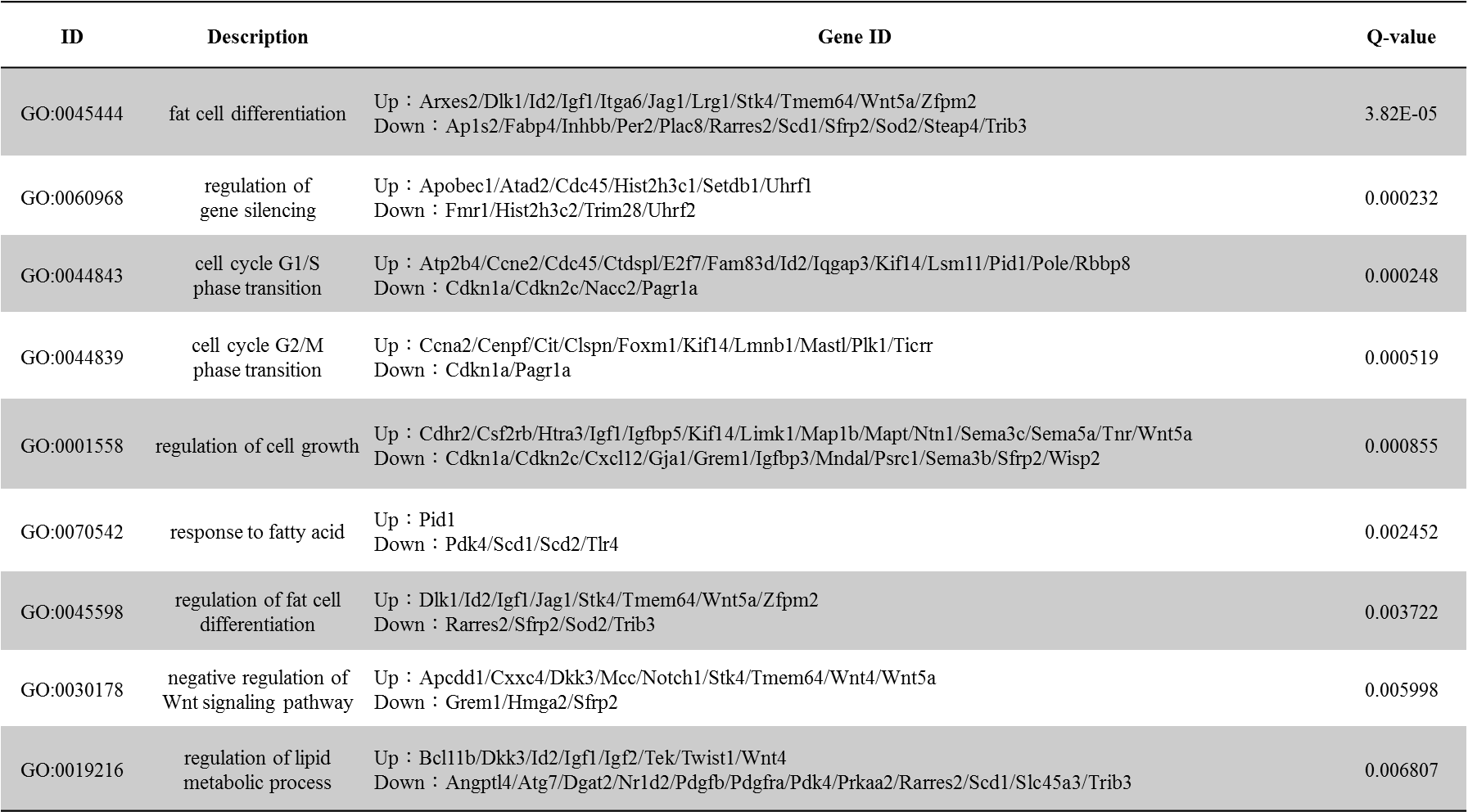


**Table S1.4 Bioinformatics analysis with Trim28 KD vs. control 3T3-L1 at day 2 by using GO database**


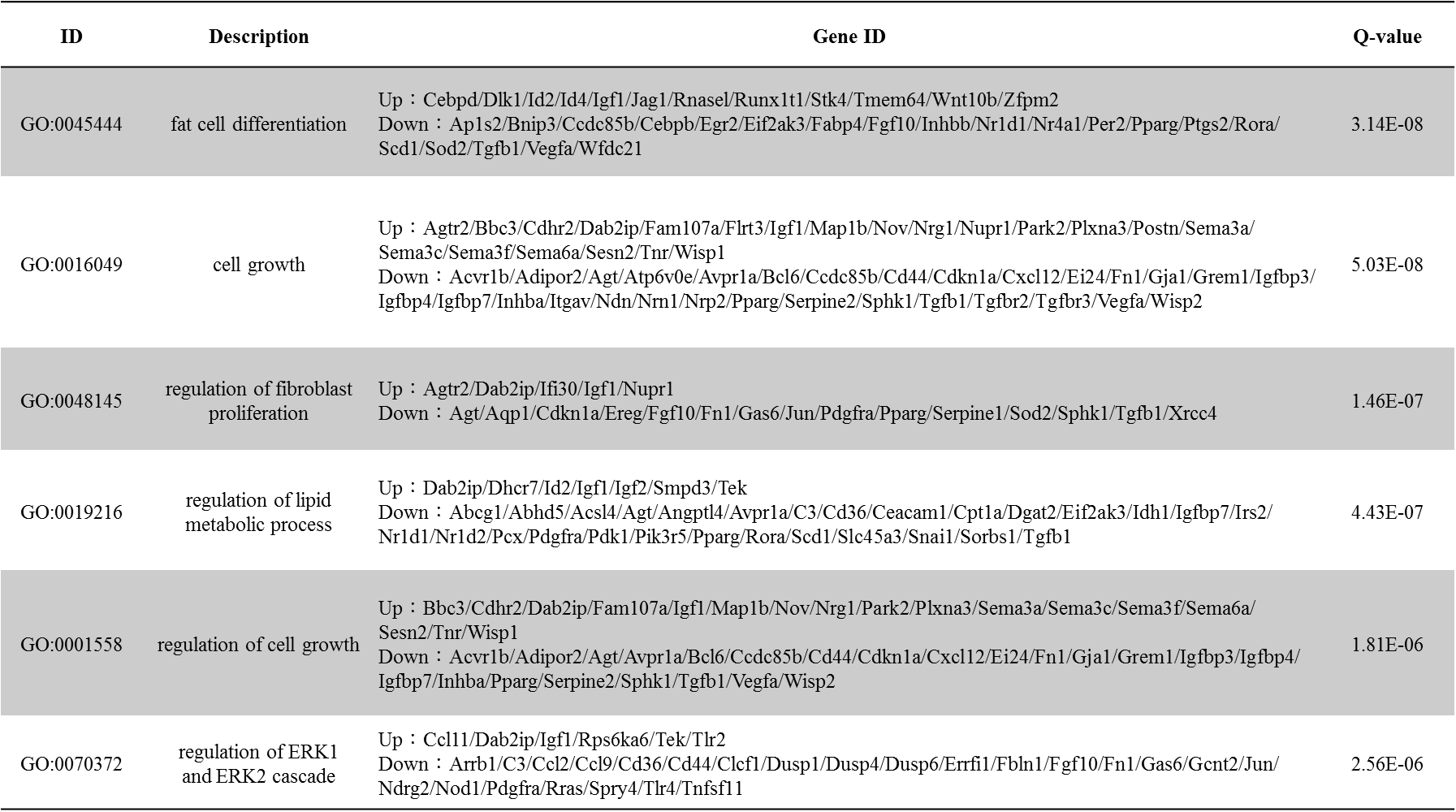


**Table S1.4 Continued**


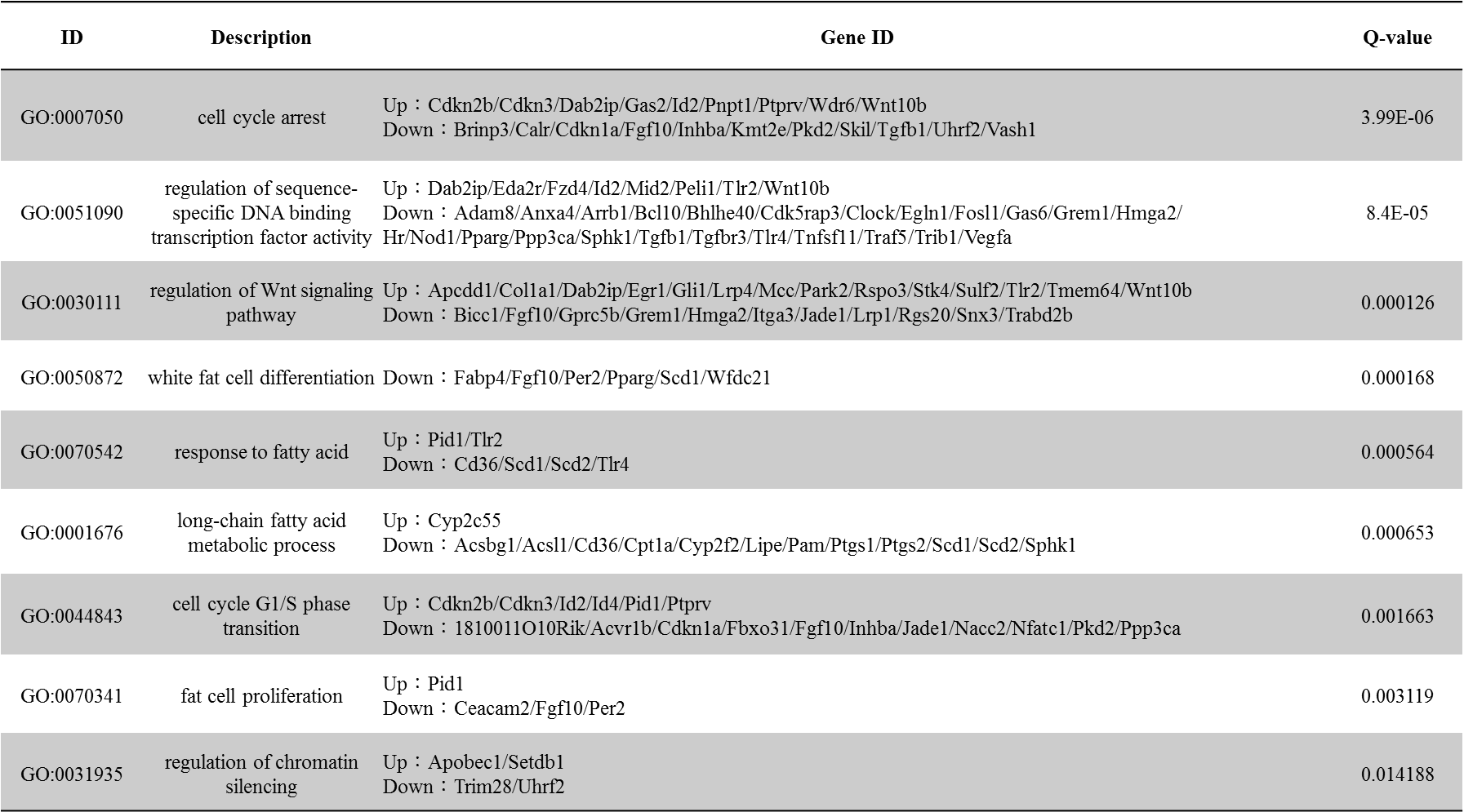

Supplement: Supplementary file 1 [file ijms-21-07245-s001.zip › Table S1.docx]
