# Supplementary material for: TRIM28 Regulates Dlk1 Expression in Adipogenesis
Source: Int J Mol Sci. 2020 Sep 30;21(19):7245. doi: 10.3390/ijms21197245 (PMC7582669; doi:10.3390/ijms21197245)
Supplement: Supplementary file 1 [file ijms-21-07245-s001.zip › TableS3.docx]

**Table S3. Primers for real-time PCR**

| Trim28 | Forward primer 5’GCACTGGACCATGACCAAAATT  Reverse primer 5’CAGAGCCCAAGAGGCAAAAC |
| --- | --- |
| β-actin | Forward primer 5’TCCTTCCTGGGCATGGAGTC  Reverse primer 5’CTCATCATACTCCTGCTTG |
| Pparg | Forward primer 5’GAAGAATACCAAAGTGCGATCAA  Reverse primer 5’GAGCTGGGTCTTTTCAGAATAATAAG |
| Cebpa | Forward primer 5’GAACAGCAACGAGTACCGGGTA  Reverse primer 5’GCCATGGCCTTGACCAAGGAG |
| Fbp4 | Forward primer 5’GATGCCTTTGTGGGAACCTG  Reverse primer 5’GCCATGCCTGCCACTTTC |
| Cebpb | Forward primer 5’GCAAGAGCCGCGACAAG  Reverse primer 5’GGCTCGGGCAGCTGCTT |
| Dlk1 | Forward primer 5’CCCAGGTGAGCTTCGAGTG  Reverse primer 5’GGAGAGGGGTACTCTTGTTGAG |
| Gapdh | Forward primer 5’AAGGTCATCCCAGAGCTGAA  Reverse primer 5’CTGCTTCACCACCTTCTTGA |
| Gtl2 | Forward primer 5’CGAGGACTTCACGCACAA C  Reverse primer 5’TTACAGTTGGAGGGTCCTGG |
