# Supplementary figures and images for: TRIM28 Regulates Dlk1 Expression in Adipogenesis
Source: Int J Mol Sci. 2020 Sep 30;21(19):7245. doi: 10.3390/ijms21197245 (PMC7582669; doi:10.3390/ijms21197245)

**Table S2. Sequences of CpG dyads at Dlk1-Gtl2 locus**

| Dlk1 promoter | 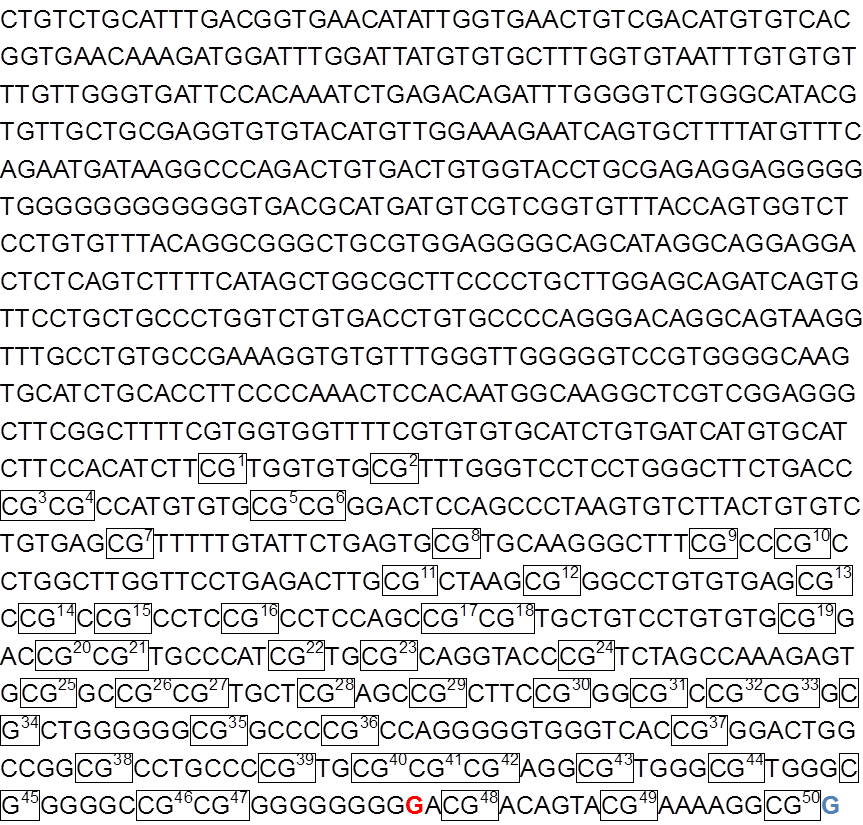 |
| --- | --- |
| Dlk-DMR | 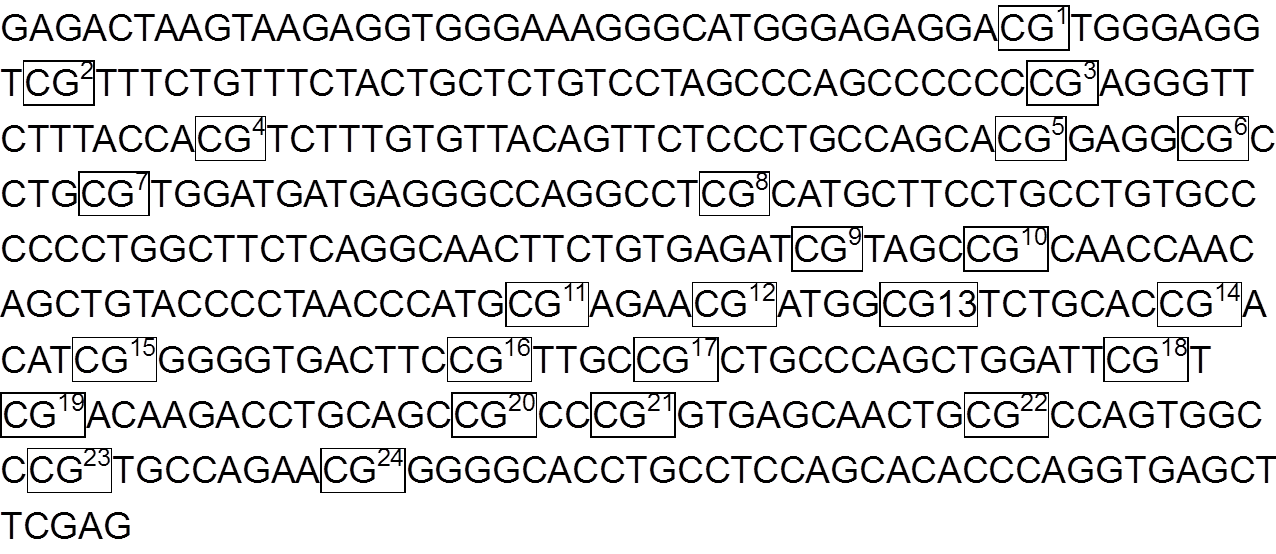 |
| IG-DMR | 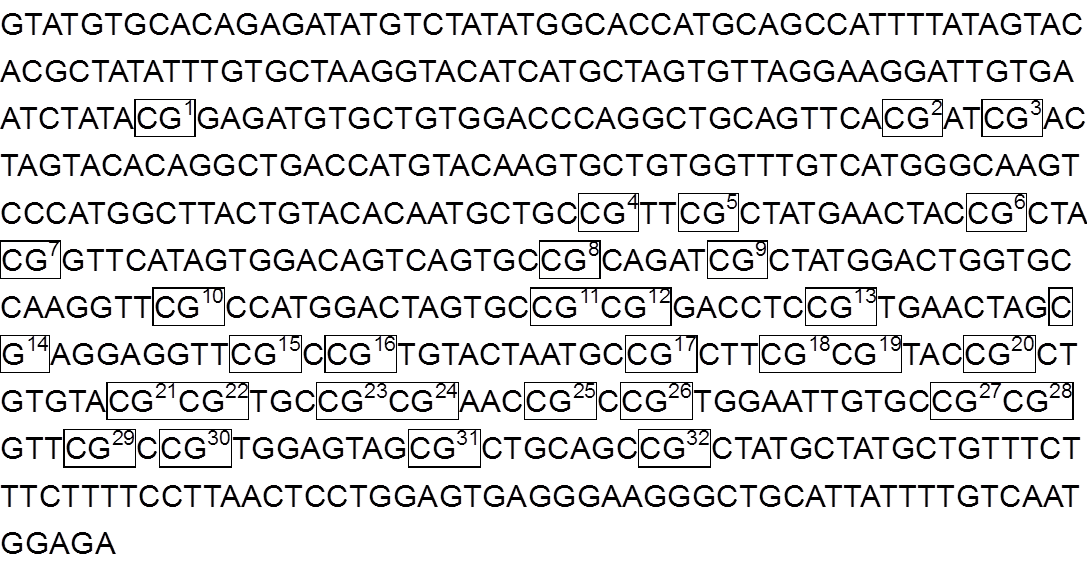 |
| Gtl2-DMR | 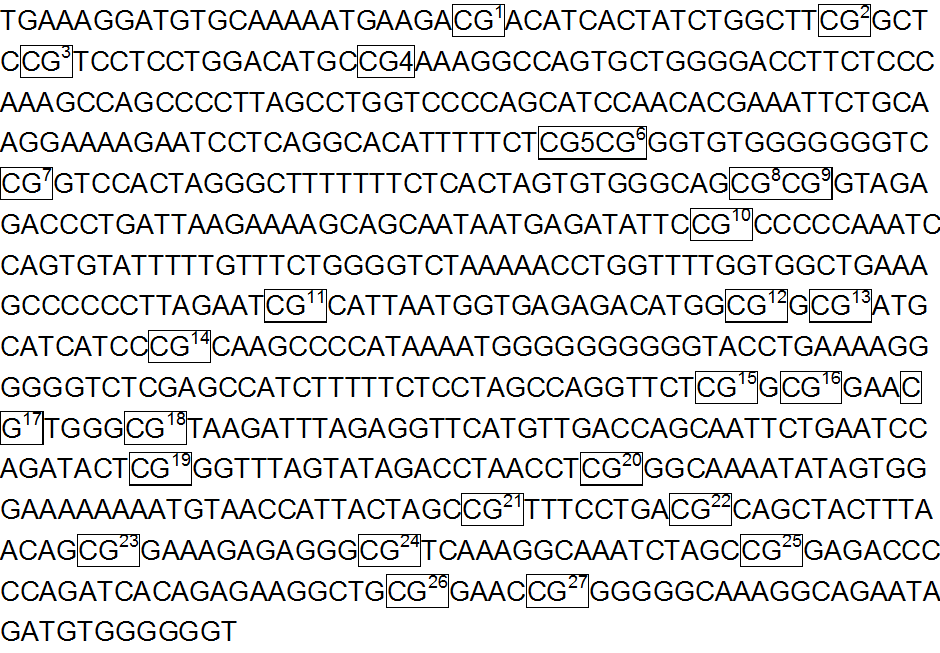 |

Supplement: Supplementary file 1 [file ijms-21-07245-s001.zip › Table S2.docx]
